# Human Milk: An Ideal Food for Nutrition of Preterm Newborn

**DOI:** 10.3389/fped.2018.00295

**Published:** 2018-10-16

**Authors:** Clair-Yves Boquien

**Affiliations:** ^1^INRA, Université Nantes, Centre de Recherche en Nutrition Humaine–Ouest, IMAD, Physiopathologie des Adaptations Nutritionnelles (UMR PHAN), Nantes, France; ^2^EMBA (European Milk Bank Association), Milan, Italy

**Keywords:** mother milk, breast milk, breastfeeding, preterm, nutritional programming, neonatal growth, infant neurodevelopment

## Abstract

Human milk is the best food for newborn nutrition. There is no ideal composition of human milk and also no easy way to control the complexity of its nutritional quality and the quantity received by breastfed infants. Pediatricians and nutritionists use charts of infant growth (weight, size, head circumference) and neurodevelopment criteria that reflect the food that these infants receive. These charts reflect first the infant physiology and likely reflect the composition of human milk when infants are breastfed. In a situation of preterm birth, mother physiology impacts partly breast milk composition and this explains how this is more difficult to correlate infant growth or neurodevelopment with milk composition. Some biomarkers (lipids, oligosaccharides) have been identified in breast milk but their function is not always yet known. A better knowledge on how human milk could act on infant development to the mid- and long-term participating thus to nutritional programming is a challenging question for a better management of infants' nutrition, especially for preterm infants who are most fragile.

## DOHAD (developmental origin of health and adult diseases) context and nutritional programming

The perinatal period is a period of organogenesis in the newborn (at the beginning of pregnancy), and of strong growth of the child during the first 2 years of life, but also in the establishment of the physiological mechanisms that persist throughout life. Since the work of Prof. BARKER (1, 2) and the formalization of the concept of DOHaD (Developmental Origin of Health and adult Diseases), it is recognized that there is a link between the conditions of development during the perinatal period and the health and adult diseases, and nutrition has a key role. It is likely that the growth rate (weight gain during the first few weeks) and the composition of this weight gain (lean body gain and body fat gain) during this key period have major long-term effects. The challenge is that the growth of the child, in this period of life, is achieved by allowing optimal neurodevelopment, without causing increased susceptibility to metabolic diseases (obesity, type-2 diabetes, and cardiovascular disease) at adulthood. And we will see later, that in prematurity of the infant, these objectives are based on nutrition conditions that are sometimes contradictory.

## Breastfeeding

In this context, the World Health Organization (WHO) recommends exclusive breastfeeding for up to 6 months, starting at the first hour of life. Despite the recommendations of the WHO and pro-breastfeeding messages delivered in hospitals and maternity hospitals, the exclusive breastfeeding rate remains quite low [even in low-income and middle-income countries, only 37% of infants younger than 6 months are exclusively breastfed (3)].

The benefits of breastfeeding, for which there is a broad scientific consensus, provide protection for the health of the infant during the first weeks of life. These are short or medium term effects:

- A highly protective effect on infant mortality, with a 12% decrease in mortality risk compared to non-breastfed (4);
- A decrease in respiratory and gastrointestinal infections during the first weeks of life of the newborn (3), probably related to the composition of colostrum (immature milk for the first 3 days of life) and breast milk that confers immune protection to the child.

Finally, there is also a consensus on the effect of breastfeeding on the improvement of neuro development (5), in children born premature (6), or on term (7). Concerning premature infants, several studies show a positive relationship between the quantity of breast milk received during hospitalization and neuro development (8). Breastfed premature children had better psychomotor development at 2 or 5 years than non-breastfed children (6), but with a slower growth (weight, height) during hospitalization, even if they caught up with non-breastfed children at 3 years of age. This was called “breastfeeding paradox” by Roze et al. (6) as previous studies have shown a positive relationship between growth rate during neonatal hospitalization and neurodevelopment (9).

All these effects are all the more pronounced as breastfeeding lasts a long time, thus highlighting a “dose” effect. However neurodevelopment advantages have been related not only to breastfeeding duration but also to the amount received, reflecting a dose response relationship (10).

Moreover, beside these benefits of breastfeeding for the child, we often forget to mention the benefits for the mother, even in the long term:

- Decreased risk of breast and ovarian cancers (3, 11);
- Decreased risk of type 2 diabetes, with a strong effect of lactation duration (12).

## Human milk composition

**Figure 1:**
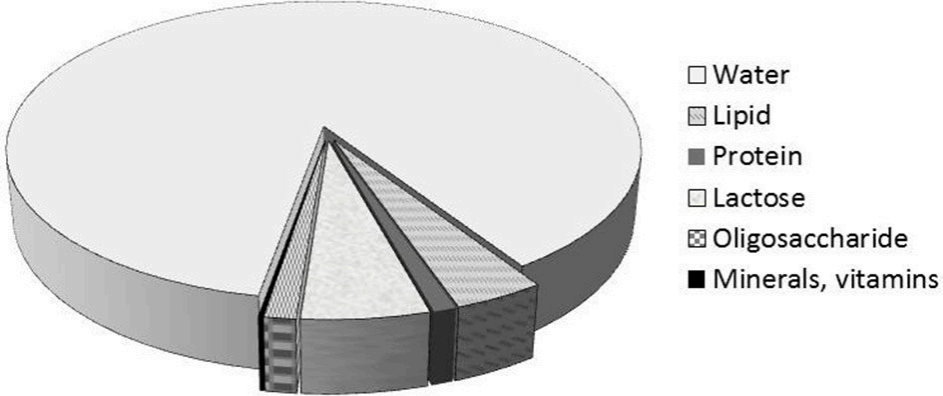
Human milk composition.

Breast milk is the best food for the newborn. Human milk consists of 87% water, 1% protein, 4% lipid, and 7% carbohydrate (including 1 to 2.4% oligosaccharides) (Figure 1). It also contains many minerals (Calcium, Phosphorus, Magnesium, Potassium, Sodium, etc…) and many vitamins. Compared to cow's milk, human milk contains less protein (3.5% in cow's milk), and especially a proportion of casein (on total protein) lower, max 50% (80% in milk of cow). There is no β-lactoglobulin; some minor proteins are more abundant in human milk (lysozyme, lactoferrin,…) and the same goes for the non-protein nitrogen fraction (urea, free amino acids, including taurine). The protein content of human milk is therefore low (10 g/L), probably the lowest among all mammalian milks, and we can relate this observation with a very low growth rate of the newborn (for comparison, rat milk has a protein content 10 times higher for a growth rate of the pups also higher).

Another peculiarity of human milk is the highest proportion of long-chain polyunsaturated fatty acids (APGI-LC), ω6 (such as arachidonic acid) and ω3 (such as eicosapentaenoic and docosahexaenoic acids [DHA]), which are derived from essential fatty acids: linoleic and α-linolenic acid. These fatty acids are important for the brain development of the infant. Compared to cow's milk, breast milk also contains more cholesterol, which is a precursor of hormones and is also involved in brain development.

Finally, oligosaccharides are present in large quantities, from 10 to 20 g/L (only 1 g/L in cow's milk) and with very varied biochemical compositions (more than 100 different compounds) (role mentioned below).

Milk contains compounds that help protect children against infectious diseases (13):

- Either by a direct immune protection, with the many immunoglobulins (including secretory IgA,…);
- Either by modulating this immune protection (by lactoferrin, pro or anti-inflammatory cytokines, or oligosaccharides);
- Either by a non-immune action, by proteins: κ-casein, α-lactalbumin, lactoferrin, haptocorrin, lysozyme (14), and oligosaccharides (see below).

Colostrum, which is produced up to 5 days after birth, also contains many immunity cells (macrophages and lymphocytes).

Milk also contains enzymes including Bile Salt Stimulated Lipase (BSSL), which allows for better lipid digestibility, and better utilization of triglycerides (95% of total lipids), and presumably LC-PUFA, cholesterol, and fat-soluble vitamins.

A certain poverty of vitamins (D and K in particular) is known, whose consequences can be avoided by a supplementation of the children, even of the mothers during the pregnancy (vitamin D). In the same way, the presence in the breast milk of chemical contaminants, highlighted for some years, raises the question of their origin, namely via the nutrition and the environment of the mother (15, 16) and their impact on the breastfed child. These contaminants accumulate in the body of the mother throughout her life because many are fat soluble and are found in adipose tissue. This is all the more pronounced since the first pregnancy of the mother is at an age more and more delayed. Preterm breast milk may also concerned by such contamination. The means to limit their presence in breast milk will be complicated to implement and will require societal, environmental and organizational policies over very long periods.

The richness of breast milk in miRNA is also one of its characteristics (17, 18). MiRNAs are non-coding RNAs that regulate gene expression and control protein synthesis at the post-transcriptional level. They play roles in the regulation of many biological and developmental processes and would be important in the development of the child's immune system. Once the milk is ingested by the child, these maternal miRNAs resist digestion, when they are protected by cellular structures (exosomes). The question of whether they are subsequently absorbed and whether they regulate genes in children is a scientific issue that is still very controversial (19).

Finally, the discovery of a microbiota of breast milk, from the 2000s, has led many teams to question its origin (endogenous entero-mammary or exogenous) and its relative role, compared to other microbiota (particularly maternal), in the colonization of the digestive tract of the newborn (20, 21). There is no complete answer to all these questions, which are complex to solve and require sampling in the most sterile conditions and not to neglect all the necessary methodological controls. Added to this is the additional difficulty of elucidating the potential interactions between all these compounds: the effect of the content of oligosaccharides of breast milk on the microbiota of the newborn is thus well studied (22), and makes it a good example of very current research.

## Physiological state of the mother impacts breast milk composition

**Figure 2:**
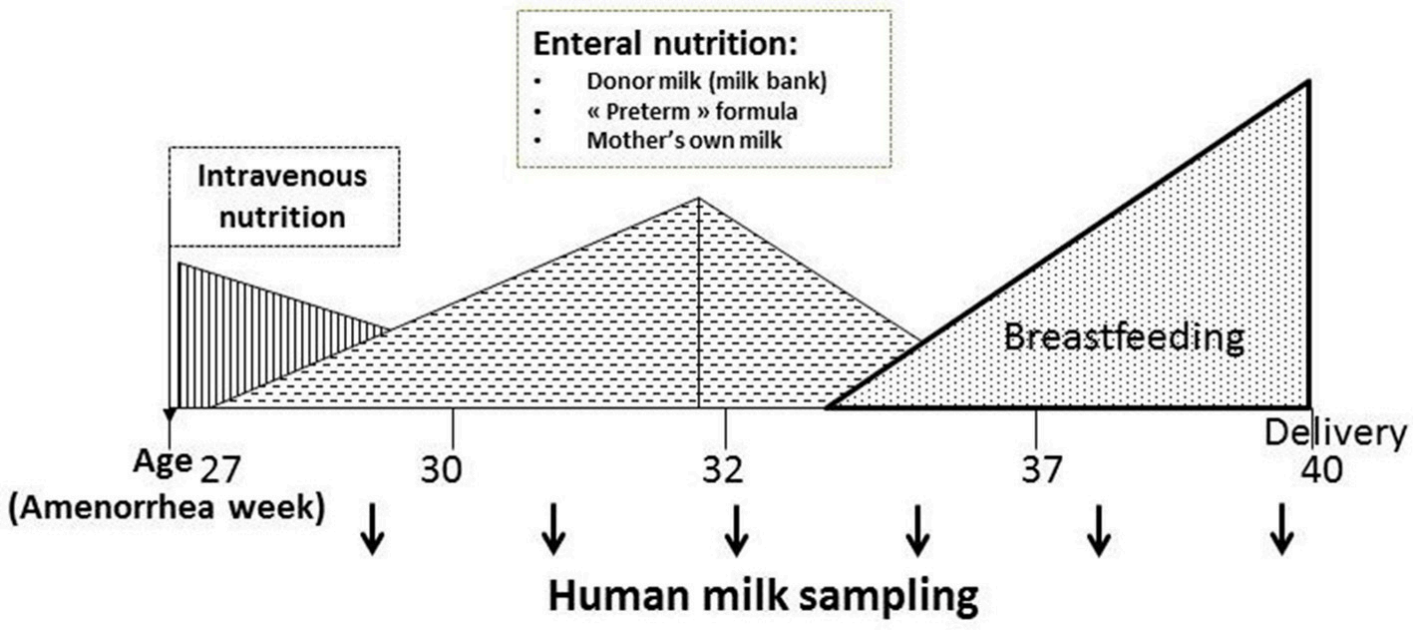
Preterm infant nutrition during early life.

The growth curve of the newborn reflects, in part, the diet (in quality and quantity) that these children receive and whether they are breastfed, presumably the composition of the breast milk they receive. But many other parameters obviously come into play, such as for example, its genetic heritage, and all the events that occur in the first weeks of the child's life (infection, etc.). We will show how it is difficult to distinguish between the effect of nutrition by breast milk and many confounding factors related to the physiological state of the mother and the clinical parameters of the child at birth (prematurity, small weight for gestational age…), knowing that some of these parameters directly influence the composition of breast milk. We can illustrate it in the following situation of prematurity:

Children born prematurely are at risk of experiencing an Extra-Uterine Growth Retardation and, as explained above, their nutritional needs are very important. Except the first days of life where they are fed intravenously (parenteral), because of their intestinal immaturity, they are quickly fed enterally with a nasogastric tube, and most often fed with breast milk (Figure 2). It turns out that the prematurity impacts the protein content of mother's milk: protein content in preterm mother's milk is higher than in term mother's milk during the first days of lactation [with maximum mean differences up to 35% (0.7 g/dl)] (23, 24) but it declines afterwards. After postnatal day 3, most of the differences in true protein between preterm and term milk are within 0.2 g/dL or less, and term milk may be the same as preterm milk by the 5th−6th week. Similarly concentration of certain free amino acids, including valine, threonine and arginine is higher in preterm mother's milk (25). Contradictory results have been obtained with lactoferrin (26, 27). Preterm breast milk appears also rich in sIgA (26, 28) and deficient in leptin (26, 29). In one study, the authors observed a decrease in serum albumin (mainly originating from blood flow) in mother's milk of infant born prematurely, which may reflect changes in the oxidative status of breast milk (30). Kunz et al. found no difference in the total amount of HMOs neither in colostrum nor in transitional or in mature milk comparing term and preterm milk samples (31) and authors list all the recent data that are consistent or inconsistent with their.

Finally, the question of whether the total fatty acid composition of mother's milk depends on the gestational age of the child remains still posed. The lower maternal-fetal transfer of fatty acids from mother to child due to a shorter pregnancy would suggest that maternal reserves of LC-PUFA are higher in mothers of premature infants and that milk such mothers would be richer in these fatty acids. However, either there is no difference with the mother's milk full-term, or the studies show quite contradictory results, except possibly for DHA (32).

## Breast milk is recommended for preterm infant feeding

**Figure 3:**
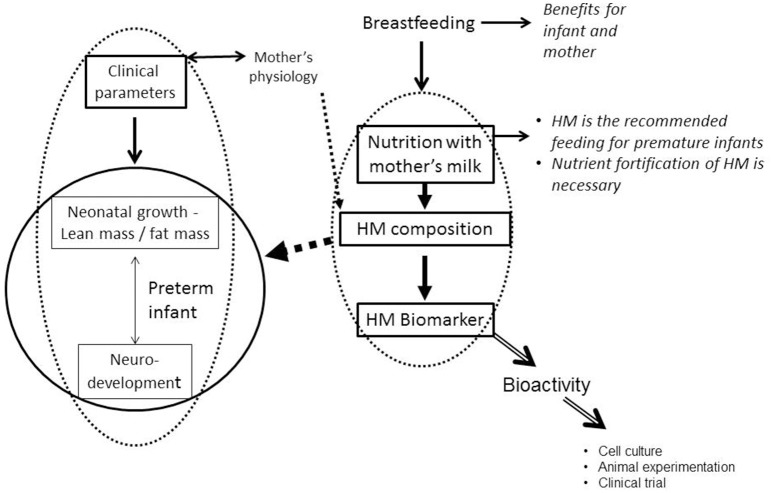
Nutrition with human milk and preterm infant growth and neurodevelopment (HM, Human Milk).

The incidence of prematurity has been steadily increasing for decades: Every year, an estimated 15 million babies are born preterm (before 37 completed weeks of gestation) throughout the world, and this number is rising. Preterm birth complications are the leading cause of death among children under 5 years of age, responsible for ~1 million deaths in 2015 (WHO, http://www.who.int/news-room/fact-sheets/detail/preterm-birth). However, progress in medical technology over the last few decades have allowed for the survival of an ever-increasing proportion of children born with very low birth weight. The challenge is therefore to improve the future of these children, and early nutrition becomes a major player in this objective. All of the expert committees recommend the use of human milk, which reduces, for example, the risk of necrotizing enterocolitis, a serious disease of premature infants in the neonatal period. However, the composition of human milk is extremely variable from one mother to another. Similarly, the long-term prognosis (in particular in terms of psychomotor development, but also in metabolic terms) is very variable among children born premature: thus, recent studies show that the development quotient remains linked to the birth term (33–36), and premature babies are at high risk of insulin resistance and metabolic disorders in adulthood (37). About 40% of premature children have psychomotor disorders at 5 years of age compared to only 12% for full-term children. A very high growth rate during this period can have deleterious effects, in terms of increased susceptibility to metabolic diseases (obesity, type 2 diabetes, cardiovascular diseases) in adulthood (38). It has also been shown that a high growth rate during the first years of life is associated with a better neuro development of the child (9). The nutrition of the child born premature thus raises many questions about the best way to do it and assessing the consequences of neonatal nutrition between susceptibility to metabolic diseases and neuro development is crucial (Figure 3).

Although breastfeeding does have a positive effect, particularly on the neuro-development of premature infants, there is great inter-individual variability in the long-term outcome. To date, very little data links the individual growth and development trajectory of a given child to the composition of breast milk received. This analysis is complicated by:

- The many biases related to breastfeeding, the particular psychological relationship between mother and child, the formation and social position of parents, the sociological determinants;
- The fortification of human milk during the hospitalization of premature infants: if breast milk is recommended for children with very low birth weight (8) and for premature infants, it brings, in the first weeks, notoriously insufficient amounts of protein per day compared to the growth needs of these newborns. All the recommendations of the European Society for Pediatric Gastroenterology, Hepatology, and Nutrition (EPSGHAN) are in line with the nutrition of premature infants by breast milk, and a protein enrichment of this milk, as soon as possible, at least until discharge from hospital, in order to increase weight gain and protein accretion (39). Most premature newborns are hospitalized during the first weeks of their postnatal life in Neonatology departments: they need support (*e.g*. respiratory) because of the immaturity of various organs and have important nutritional needs to cover their high energy expenditure in addition to growth during this period. But the debate is lively between neonatologists on the methodology to follow. This topic is debated in an article in the same collection “Human Milk in the Feeding of Preterm Infants: Established and Debated Aspects” (Arslanoglu et al., manuscript under revision). Then, behind this debate between “standardized” vs. “individualized” fortification, there is a low level of knowledge about the variability of the composition of breast milk (micronutrients in particular), from one mother to another, and according to the lactation time, and the consequences on the growth and development of the child, in the short term and even in the medium term.

## Relationship between breast milk composition and growth and neurodevelopment of infant

If breast milk has certain plasticity in its composition, depending on the physiology of the mother, does this affect the physiology of the breastfed child, its growth trajectory, or even its neurological development (Figure 3)? The answers to this question are to be found both in experimental researches on animal models and in clinical research, in retrospective studies, which present a longitudinal follow-up of breastfed children.

The breastfed infant receives nutrients from the mother's milk, which, once hydrolyzed or not, passes the intestinal barrier and ends up in the blood. It is therefore normal for the blood metabolome or lipidome to be different between a breastfed child and a non-breastfed child. So at 3 months, the blood lipidome of children exclusively breastfed for 3 months is very different than receiving an infant formula (40), with differences to phosphatidylcholines, sphingomyelins, and triglycerides. This result is not necessarily linked to the difference in triglyceride composition between breast milk and infant formula but it may also be that the neonatal nutrition have had additional effects on lipid metabolism, which substantially modified the lipidome of the infant at 3 months.

The issue of breast milk in nutrition programming is really difficult to solve. In fact, the inter-individual variability of human milk, and the heterogeneity of breastfeeding times (dose effect) complicate the associations between a particular composition of breast milk and certain clinical parameters of the child who has received this milk. Among the retrospective studies that have addressed this issue, lipids and oligosaccharides in breast milk, as well as some micronutrients, have been the subject of recent publications.

Lipids in human milk are the second most important macronutrient in breast milk, and have been studied extensively since the 2000s (41–46). Human milk is rich in linoleic acid (LA) and α-linolenic acid (ALA) which are the precursors of long-chain polyunsaturated fatty acids (PUFAs) ω6 and ω3; these precursors are not synthesized *in vivo*, and breast milk is the only source of intake for the breastfed child. These PUFAs are essential for brain development, including DHA; breast milk is also rich in DHA and so it brings both DHA and its precursor ALA. As brain growth continues during the child's first weeks of life, especially for the premature infant, this intake of ALA and DHA is essential.

Associations have been sought between breast milk lipids and growth and development of the child. Several studies concern term infants (47, 48) but the development of preterm infants has also been studied. Mead acid (C20:3 n-9, an omega-9 fatty acid) in early breast milk is associated with general movement's score at 40 weeks of gestational age suggesting that increased concentration in mead acid influenced this score negatively (49). Similarly arachidonic acid was also negatively correlated with some behavioral assessment scores. These relatively simple associations reflect likely a more complex reality such as a shortage of fatty acids ω6 and ω3 or an imbalance between ω3 and ω6 fatty acids.

To better understand the role of different families of breast milk lipids, in a context of prematurity, we conducted a pilot study, on 2 groups of 11 mothers of infants born premature, and selected in the LACTACOL cohort (NCT01493063), on the basis of growth (rather good [difference between discharge and birthweight Z-score during neonatal hospitalization of −0.5] vs. rather poor [difference between discharge and birthweight Z-score during neonatal hospitalization of −1.5]) of their breastfed children. We found a strong relationship between the growth of these premature children during hospitalization and the presence of several lipid biomarkers in breast milk, identified by both targeted methods of fatty acid assays and non-targeted methods without *a priori* (lipidomic analysis). Based on the lipidomic profiles of breast milk, obtained after analysis by liquid chromatography coupled with mass spectrometry, and after using discriminating statistical tools, several lipid species have been selected for their ability to predict the weight growth of premature infants, during their first 4 weeks of life. The faster growth was associated with milk containing more medium chain saturated fatty acids and sphingomyelin, more phosphoethanolamine containing dihomo-ɤ-linolenic acid, and less oxylipines (50).

The oligosaccharides of human milk are present in high concentration (51–53) and have many functions:

- They have a “prebiotic” effect (54, 55) and can therefore be considered as non-digestible dietary components that beneficially affect the health of the host by selectively stimulating the colon, growth and/or activity of a species or a limited number of bacterial species (56). Several recent studies have shown a link between the presence of oligosaccharides and the microbiota of the newborn (22, 57–60).
- They participate in the inhibition of bacteria, viruses or even parasites (22, 61): by the similarity of their structure with the receptors present on the intestinal mucosa, they play a role of decoy, on which are fixed bacteria and viruses. Many pathogens use lectins to attach to the glycans of the intestinal epithelium. Human oligosaccharides have structures close to those of cell surface glycans and pathogens bind with oligosaccharides instead of surface glycoproteins / glycolipids…but a human oligosaccharide cannot block all lectins alone!
- They would modulate certain immune reactions because certain human oligosaccharides interfere *in vitro* with cell-cell interactions mediated by selectins;
- They are rich in sialic acid found in brain glangliosides (62);
- They protect the premature newborn against necrotizing enterocolitis (NEC) (63, 64).

The oligosaccharides fraction of preterm breast milk is likely the most interesting one raising challenging scientific questions. This is due to several reasons:

- The increase in oligosaccharides diversity over time with 56 HMO present in mature milk at 40 weeks of post-menstrual age that were not present at birth (65) revealing a new aspect in immaturity of preterm human milk at the beginning of lactation with likely some consequences on infant's gut colonization;
- The presence of an α 1,2 linked fucosylated HMO, 2′ fucosyllactose (2′ FL) in human milk indicates that the mother is a secretor and 60 to 80% women are secretors. De Leoz et al. found an unexpectedly high number of apparent non-secretors among women delivering preterm and the lack of consistency in “milk secretor status” over time in a few women (65) with momentary strong decline in 2'FL concentration. However, these conclusions need to be confirmed by studies with larger sample size;
- The relationship between a secretor status and a protective effect against bacterial dysbiosis defined as a delayed maturation of infant microbiota and against NEC (66);
- The microbiome of children with large growth delays is not refractory to nutritional supplementation with oligosaccharides (67, 68). This opens interesting perspectives for the care of preterm infants.

Other milk compounds have also been tested for their ability to predict the clinical characteristics of the breastfed child but mainly for term infants [fructose (69), leptin (70), TNFα et IL6 (71)]. A very recent review completes all of these data (72). The difficulty of all these studies is that many of them are only proof of concept studies with small size samples that will need complementary trials.

In preterm infants the overall breast milk composition impacts the gut microbiota with a greater bacterial diversity and a more gradual acquisition of diversity in infants fed breast milk compared to infants fed infant formula (73). This could be explained by the presence in human milk of oligosaccharides, of a microbiome and of secretory IgA that protect infant against pathogenic bacteria. Since the developmental pattern of preterm infant microbiota is characterized by different phases (74), the association between macronutrient intake and growth appears very complex depending on the composition of the gut microbiota and differing between microbiota phases. This opens new opportunities to fortify human milk differently for each preterm infant in a precision medicine.

## A biomarker in breast milk does not necessarily mean a bioactivity

All previous work demonstrates biomarkers of growth of the newborn in breast milk, and those we have described are essentially oligosaccharides or lipids. But a biomarker does not necessarily have a biological activity because it can only represent the product of a metabolism. To prove its bioactivity, it is necessary to use a cellular model (to test an activity in cell culture, including cell proliferation, differentiation, secretion of cytokines, or the expression of growth factors, etc.), animal experimentation or finally the clinical study (Figure 3): an infant formula is supplemented with the biomarker to test its bio-activity, for example its effect on the growth of the newborn. Many minor proteins, present in breast milk and absent in cow's milk, or present in high concentration compared to cow's milk, have been tested. This is the case of lactoferrin, which has antimicrobial activity (75), which acts on the absorption of iron (76) and is bifidogenic (77). Oral lactoferrin supplementation decreases late-onset sepsis, NEC, and “all-cause mortality” in preterm infants without adverse effects but authors conclude that the evidence is moderate- to low-quality (78).

Demmelmair et al. (14) recently identified these minor proteins that have been the subject of clinical studies:

- Lysozyme with a 1000 times higher concentration in human milk than in cow's milk;
- Osteopontin which is 10 times more concentrated in human milk than in cow's milk and which plays a role in the immunity of the child;
- Bile salt stimulated lipase, present in human milk, which would improve the digestibility of long-chain fatty acids. In a multi-center randomized trial, recombinant BSSL has been tested vs. placebo with a follow-up for 12 months; there was no improvement in weight growth in preterm infants except in the sub-group of Small for Gestational Age infants but with some imbalance in the adverse effects (79).
- α-lactalbumin, which improves the absorption of iron;
- Lactoferrin, about 20 times more concentrated in human milk compared to cow's milk, and whose functions have been mentioned above.

In addition to these minor proteins, a complex of proteins and lipids has been the subject of several clinical trials: these are Milk Fat Globule Membranes (MFGMs). The fat globules are surrounded by a triple membrane which is a complex construct. The outer membrane is hydrophilic and allows the dispersion of fat globules in milk which is an oil-water emulsion. These membranes contain cholesterol, glycerophospholipids, sphingolipids, and minor proteins (mucin 1, xanthine oxidoreductase, butyrophilin, lactadherin, adipophilin)(14). About 25–70% of these MFGMs are proteins. Infantile preparations enriched with MFGM or constituents of these MFGMs (phospholipids) have been tested in clinical trials with convincing results in terms of programming of immunity and cognitive functions.

These differences in composition between human and bovine milks highlight that human milk composition is very specific and certainly better adapted for preterm infants. Even if formula producers have improved their process to better mimic human milk, large discrepancies will continue to exist.

## Conclusions

Breast milk is a “natural” and “sustainable” food, without any impact on the environment, with a strong symbolic value, since it represents the first “vector” of transmission of taste, rules governing the terms of the meal, and so of the food identity (80). It is a reference for the nutrition of the newborn, even if there is no ideal composition. It is best adapted to the nutrition of the newborn and breastfeeding is not recommended in a few rare diseases [in case of infection with HIV (AIDS virus), except in countries where access to infant formulas and especially to drinking water is difficult, and in case of galactosemia, a disease that does not allow the child to metabolize galactose]. The benefits of breastfeeding have been quantified economically and increased breastfeeding rates bring substantial savings for any health system (81).

Benefits of breastfeeding are recognized and although it must be admitted that breast milk is not always perfect, the benefit or risk balance is leaning on the benefits side, especially for preterm infants. The scientific consensus is that breast milk is the best food for preterm infants as soon as their digestive maturity allows them to digest proteins and lipids. In fact preterm mortality has decreased a lot during the past years because these infants are really better taken care in neonatology units. Better managing nutrition of these infants would likely improve their development. In order to improve milk fortification, we need to better know the complexity of nutritional composition and the relationship between this composition and infant physiology.

Although it contains macronutrients with a fairly stable concentration, breast milk has a very plastic micronutrient composition, which depends in particular on the physiology of the mother. The question of whether it has an adaptive composition, according to the needs of the child, remains to this day unanswered.

## Author contributions

The author confirms being the sole contributor of this work and has approved it for publication.

### Conflict of interest statement

The author declares that the research was conducted in the absence of any commercial or financial relationships that could be construed as a potential conflict of interest.

## Abbreviations

ALA: α-Linolenic Acid
BSSL: Bile Salt Stimulated Lipase
DHA: DocosaHexaenoic Acid
HMO: Human Milk Oligosaccharide
IUGR: Intra-Uterine Growth Restriction
LA: Linoleic acid
LC-PUFA: Long Chain PolyUnsaturated Fatty Acid
MFGM: Milk Fat Globule Membrane.
